# Common Roadblocks for Biomaterials Metrologists

**DOI:** 10.3390/jfb7020012

**Published:** 2016-05-18

**Authors:** Michael Wininger

**Affiliations:** 1 Prosthetics & Orthotics Program, University of Hartford; West Hartford, CT 06117, USA; wininger@hartford.edu; Tel.: +1-860-768-5787; 2 Department of Biostatistics, Yale School of Public Health; New Haven, CT 06520-8034, USA; 3 Cooperative Studies Program, Department of Veterans Affairs, West Haven, CT 06516, USA

In this issue, Naylor *et al.* [1] report on the surface topography of prosthetic phalanges, important research that is increasingly vital to researchers and clinicians alike. While their approach is within the conventions of the field, and therefore cannot be impugned, this publication evidences common roadblocks for biomaterials metrologists.

Naylor *et al*.’s paper (*J. Funct. Biomater.* **2016**, *7*, 9, doi:10.3390/jfb7020009) looks at a single specimen per phalanx; this is an inherent limitation, and speaks to the utter difficulty in establishing adequate sample size for estimating error and asserting homogeneity and repeatability. Naylor *et al.*’s paper also poses two suggestions about the putative impact of polishing and lubrication on these implants; where these conjectures are unaccompanied by supporting justification, e.g., demonstration of scale compatibility between a polishing tool and lubricant at the surface, shows that there remains much to be learned about the physicochemical stresses at the biomaterial interface.

Lastly, we note the use of surface roughness measures root-mean-square (RMS), skewness and kurtosis. These parameters measure the variability of feature height across the sample surface relative to the arithmetic mean. These methods were not invented by Naylor *et al.*; rather, these measures have been promulgated over the past 35 years by institutions of the highest repute in governmental and industrial standards-making, including the NIST [2], NASA [3], the National Physical Laboratory-UK [4], and the British Standards Institute (ISO-7206-2) [5].

This population-average approach is limited by the lack of spatial information. Roughness is an inherently ”neighborly: concept, where “smooth” reflects a small height difference between adjacent loci, and “rough” reflects a large difference in height between adjacencies. To account for spatial relationships, topographic measures should account for spatial variables. As an example, we consider the conventional measures as posed in Naylor *et al.*, e.g., skewness:

(Skewness) $$\delta \propto \underset{X}{\int }\underset{Y}{\int }{\left(\frac{z\left(x,y\right)-\mu }{\sigma }\right)}^{3}\;dx\;dy$$

where *z* is the surface height measured at *x* ϵ *X* and *y* ϵ *Y*, and note its spatial insensitivity: the integrand considers only the global parameters μ and σ: the global mean and standard deviation of feature heights across the sample. Now consider a modest re-formulation that incorporates height differences between adjacent loci:

(Roughness) $$\rho \propto \underset{X}{\int }\underset{Y}{\int }\left({\left(\frac{d^{3}}{dx^{3}}z\left(x,y\right)\right)}^{2}+{\left(\frac{d^{3}}{dy^{3}}z\left(x,y\right)\right)}^{2}\right)\;dx\;dy$$

where $\frac{d^{3}}{dx^{3}}z\left(x,y\right)$ and $\frac{d^{3}}{dy^{3}}z\left(x,y\right)$ are the instantaneous rate of change of the “acceleration” of feature height *z* with respect to spatial variables *x* and *y*. This is the spatial analogue to the jerk common to researches in robotics, biomechanics, and motor control [6,7].

We show the performance of skewness δ and roughness ρ on two simulated surfaces with clearly distinguishable roughness profiles (Figure 1). Consider a completely smooth surface (upper panel) and the same surface with all loci randomly reorganized (lower panel). The population average (and therefore skeweness) is identical in both images, but the spatially sensitive roughness is starkly different.

It is only fair to view Naylor *et al.*’s approach in the context of established practices. Additionally, for that matter, prudence must be used in applying jerk to a dataset, as whether and how to normalize jerk remains an open line of inquiry [8,9,10]; even the most theoretically sound approach requires validation and revision through implementation. Nevertheless, research enterprise in metrology must adhere to the same empirical best practices expected elsewhere in science: using adequate sample size and proper statistical design, a well-posed analytical approach, and scrutable interpretations. It should be strongly encouraged that studies based on a single specimen be withheld until additional samples can be collected and analyzed, and that investigators refrain from unsupported speculation. In particular as it concerns material science, we must immediately revoke the use of distribution-based measures in characterizing spatially sensitive phenomena (*i.e.*, RMS, skewness and kurtosis as a measure of surface roughness), and revisit conclusions drawn from extant studies where these flawed practices were adopted. Indubitably, we should advise the standard-makers to revise their guidance documents so as to eliminate the error at its source.

## Figures and Tables

**Figure 1:**
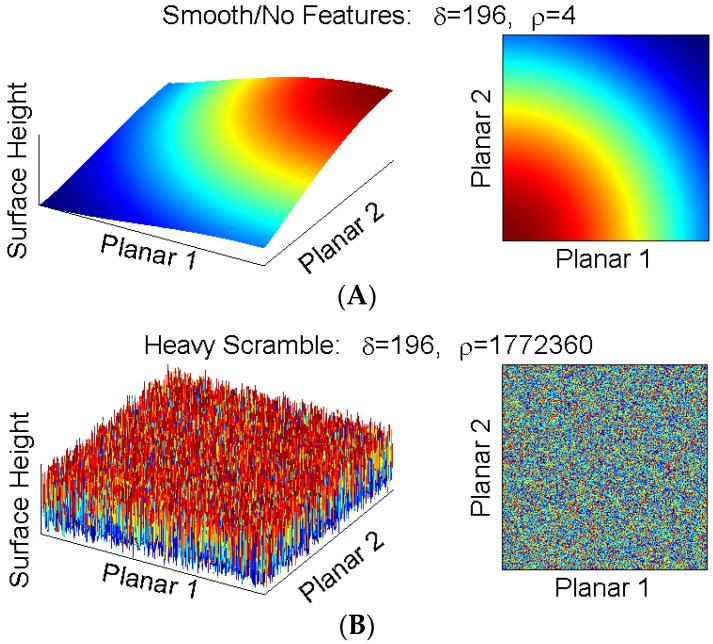
Skewness (δ) and roughness (ρ) for two simulated surfaces. Smooth surface (**A**) and smooth surface randomly re-configured (“scrambled”) into a rough surface (**B**). Both surfaces contain the same set of pixels; consequently, δ remains the same in both samples (sample mean μ and standard deviation σ do not change). However, the height differences between adjacent loci are captured via use of the derivative in ρ; as a result, the calculated roughness matches the visual profile.
